# Implementation of Computer-delivered Brief Alcohol Intervention in HIV Clinical Settings: Who Agrees to Participate?

**DOI:** 10.4172/2155-6105.1000276

**Published:** 2015-04-10

**Authors:** Cui Yang, Heidi M Crane, Karen Cropsey, Heidi Hutton, Geetanjali Chander, Michael Saag, Mary E McCaul

**Affiliations:** 1 Department of Health, Behaviour and Society, Johns Hopkins Bloomberg School of Public Health Baltimore, USA; 2 Department of Medicine, University of Washington Seattle, USA; 3 Department of Psychiatry and Behavioural Neurobiology, University of Alabama at Birmingham, USA; 4 Department of Psychiatry and Behavioural Sciences, Johns Hopkins University School of Medicine Baltimore, USA; 5 Department of General Internal Medicine, Johns Hopkins University School of Medicine Baltimore, USA; 6 Department of Medicine, University of Alabama at Birmingham, USA

**Keywords:** Alcohol misuse, Brief intervention, HIV, Mental health, Implementation

## Abstract

**Objective:**

Addressing alcohol use in primary HIV settings can improve medical outcomes and overall quality of life of persons living with HIV (PLWH). In order to assess the feasibility of computer-delivered brief alcohol intervention (CBI) and to inform future efforts to improve access to CBI, we examined patient-level socio-demographic, clinical and behavioral characteristics associated with agreement to participate in CBI among non-treatment seeking PLWH with alcohol misuse.

**Methods:**

Participants were recruited from two Centres for AIDS Research (CFAR) Network of Integrated Clinical Systems (CNICS) HIV clinics. PLWH completed a clinical assessment of patient-reported measures and outcomes using tablet-based assessments, including socio-demographic and behavioural characteristics. HIV biological indicators, i.e., CD4 count and viral load, were also available from the electronic medical record. Participants were approached for CBI participation based on scores on the Alcohol Use Disorders Identification Test (AUDIT); no incentives were offered for CBI participation. We performed chi-square tests, analysis of variance and multivariate logistic regression to compare socio-demographic, behavioural and clinical factors among participants who agreed to participate compared with those who refused/postponed participation.

**Results:**

We observed that 42% of non-treatment seeking, non-incentivized PLWH with alcohol misuse provided written agreement to participate in on-site CBI delivered in their HIV primary care clinic. A larger proportion of PLWH who agreed to enrol in CBI had detectable viral loads, heavier weekly alcohol use, and higher DSM-5 alcohol use disorder symptom counts and mental health symptoms. Neither socio-demographic background nor drug use status was associated with CBI enrolment.

**Conclusion:**

CBI implementation reached those patients most in need of care. The findings of this study may assist HIV-care providers to better identify appropriate patients and initiate discussions to facilitate the participation of PLWH in alcohol intervention services.

## Introduction

Prevalence of alcohol misuse (heavy/binge drinking and alcohol use disorder) is elevated among persons living with HIV (PLWH) compared with the general population [1–3]. PLWH who are heavy/binge drinkers may be more likely to engage in risky sexual behaviors and thereby increase rates of HIV transmission [4]. Research has demonstrated that alcohol misuse negatively affects every step of the HIV treatment cascade, including delaying testing for infection, accessing appropriate medical care, initiating antiretroviral therapy (ART), and ART non-adherence [5]. Furthermore, alcohol misuse contributes to many comorbid conditions that may impact progression of HIV infection, such as among patients with HIV/HCV co-infection [6].

Given the deleterious relationship between alcohol misuse, HIV transmission and outcomes, addressing alcohol use in HIV primary care settings is essential to improve the overall quality of life and long-term medical outcomes of PLWH. HIV clinics offer an excellent opportunity for the integration of a brief alcohol intervention (BI), given the long-term care of patients, the need for regularly scheduled follow-up appointments, and intensive case management models that promote outreach to and retention of patients who are often challenging to treat. BI has been shown to be effective in decreasing alcohol use in general medical patients [7] and PLWH [8]. Despite considerable efforts to encourage providers to adopt BI in practice, various implementation barriers exist in primary healthcare settings, including a reluctance of patients to disclose alcohol misuse to their healthcare providers, limited financial resources in clinics, and a significant time commitment from providers [7].

Over the past decade, there has been increasing interest in the development of computer-delivered alcohol brief intervention (CBI). CBIs have been designed with the goal to overcome some provider-delivered BI implementation barriers; they are low cost, fully reproducible, nonjudgmental, pose less burden on primary care providers and present no need for extensive training [7]. In addition, CBI can be tailored to the unique needs of PLWH with a chronic and stigmatized disease by providing greater confidentiality and flexibility of program content and branching [9].

We recently introduced CBI in two HIV primary care clinics as part of an implementation study to disseminate evidence-based alcohol therapies to reduce alcohol misuse in PLWH. The intervention is a two-session, 12–15 minute computer-delivered motivational intervention offered to individuals with alcohol misuse based on elevated AUDIT-C scores. In order to assess the feasibility of CBI and to inform future efforts to improve access to CBI, we sought to examine patient-level socio-demographic, clinical and behavioural characteristics associated with agreement to participate in CBI among non-treatment seeking PLWH with alcohol misuse.

## Methods

Participants were recruited from Centres for AIDS Research (CFAR) Network of Integrated Clinical Systems (CNICS) HIV clinics in Seattle, WA and Birmingham, AL. PLWH completed a clinical assessment of patient reported measures and outcomes using tablet-based assessments at the time of routine clinic appointments. In addition to socio-demographic characteristics (i.e., sex, race, age), assessments included recent alcohol use (Alcohol Use Disorders Identification Test consumption items (AUDIT-C)) [10], illicit drug use (Alcohol and Substance Involvement Screening Test (ASSIST)) [11], depressive symptoms (Patient Health Questionnaire (PHQ-9)) [12], panic symptoms (PHQ panic symptom scale) [13] and an antiretroviral medication adherence rating scale [14]. Patients with elevated AUDIT-C scores completed the full AUDIT and the M.I.N.I-International Neuropsychiatric Interview 7.0 alcohol section [15] on the tablet to assess for alcohol-related problems and alcohol use disorder (AUD). HIV biological indicators i.e., CD4 count and viral load, were also available from the electronic medical record.

Inclusion criteria for CBI were an AUDIT-C score > 4 if male, > 3 if female; 18 years or older; and English-speaking. The clinical assessment platform automatically notified the Research Assistant in real time by pager when a patient was eligible. Using a recruitment script, the Research Assistant provided a brief description of the study and invited the patient to provide written informed consent. The recruitment script emphasized that CBI may help patients reduce alcohol use and CBI will be brief and integrated with patient’s regular clinic visits. The Research Assistant made clear that the patient’s decision on participation in CBI would not affect his/her care in the HIV clinic. Patients remained eligible for study enrolment for one year once they met study eligibility requirements. The study was approved by the local Institutional Review Board at each site. No incentives were offered for study participation as this was an implementation study to see how CBI could be integrated within routine clinical care.

### Statistical Analyses

The primary outcome of interest for these analyses is written agreement to participate in CBI. Independent variables were sex, race, age, CD4 count, HIV viral load, medication adherence, alcohol use severity (AUDIT-C score), M.I.N.I. AUD symptoms count, presence or absence of illicit drug use, cigarette use, panic and depressive symptom severity. We performed chi-square tests and analysis of variance (ANOVA) to compare socio-demographic, behavioural and clinical factors among participants who agreed or refused/postponed participation. Variables that were significantly associated with the outcome of interest (p < 0.05) were used in bivariate and multivariate logistic regression models. We used multiple imputations to handle missing data. All analyses were performed using Stata Version 13.0 [16].

## Results

Between June 2013 and August 2015, 550 individuals were approached to participate in CBI, of whom 230 agreed (42%), 214 refused (39%) and 106 (19%) postponed enrolment over the one-year eligibility window. The current analyses compared patients who agreed to enrol (n = 230) with those who either refused participation or postponed enrolment beyond their one-year eligibility window (n = 320). Socio-demographic, clinical and behavioural characteristics of participants and their associations with CBI enrolment are presented in Table 1. The majority of individuals were male (82%) with an average age of 43, 48% were black, 44% were white, and 8% were Hispanic and other races.

Individuals who agreed to enrol in CBI reported heavier weekly alcohol use (F = 4.84, p = 0.03, df = 539) and had higher DSM-5 AUD symptom counts on the M.I.N.I. (F = 6.95, p = 0.009, df = 462). We also observed a trend that a larger proportion of participants who agreed to participate reported seven or more drinks on a typical day of drinking compared to refusers or postponers (*X*^2^ = 5.44, p = 0.07). Patients who agreed to enroll also had more severe mental health symptoms than those who refused or postponed participation. Specifically, a larger proportion of CBI enrollers reported higher scores on the PHQ panic symptom scale indicating probable panic disorder or/and mild, mild-moderate, moderately severe or severe depressive symptoms on the PHQ-9 (*X*^2^ = 11.05, p = 0.001). Finally, a higher proportion of CBI enrollers had a detectable viral load on their most recent laboratory test compared to those who refused or postponed (*X*^2^ = 4.07, p = 0.04). Neither socio-demographic background variables (i.e., age, sex or race) nor drug use were associated with agreement to participate in CBI.

In the adjusted model (Table 1), undetectable viral load (adjusted odds ratio [aOR]: 0.67, 95% Confidence Interval [CI]: 0.44, 1.00) and mental health symptoms (aOR: 1.67, 95% CI: 1.16, 2.39) remained marginally significantly or significantly associated with CBI enrollment.

## Discussion

In this implementation study of CBI, more than 40% of non-treatment seeking PLWH with alcohol misuse agreed to participates. Notably, patients were approached in the context of their regularly scheduled medical visit, had not solicited help for their drinking and were not offered any incentives for CBI participation. Uptake of alcohol BI in previous studies across different settings, including community pharmacies [17], emergency departments [18] and primary care clinics [19], has varied from 21% to 45%. We found that a higher proportion of CBI enrollers had higher severity of alcohol misuse, mental health concerns (i.e., panic symptoms and moderately serve or severe depressive symptoms), and advanced HIV disease progression (i.e., detectable viral load), indicating that CBI implementation reached those most in need of care. Our findings are consistent with previous studies that found patients’ alcohol misuse severity was an influential determinant of BI participation [20]. Results also are in line with earlier reports that comorbid depression and anxiety disorders increase the likelihood of engagement in alcohol treatment services [21]. The findings of this study may assist HIV-care providers to better identify appropriate patients and initiate discussions to facilitate the participation of PLWH in substance use services. More research is needed to understand how to best tailor alcohol services to different patient populations and realize the potential benefits of integrating existing evidence-based approaches into HIV care clinical settings.

Limitations of this study should be noted. The first limitation is generalizability. Patients were recruited from two clinics and findings may not necessarily be generalizable to other clinical populations and settings. Despite this limitation, participants in this sample represent similar socio-demographic background as the population living with HIV/AIDS in the United States [22]. Secondly, the study relied on participants’ self-reports of their alcohol and illicit drug use behaviors which are subject to recall and social desirability bias. However, self-reported alcohol use generally underestimates actual drinking quantity and frequency, so it is likely that alcohol consumption was actually higher than self-reports. Finally, sample size may have limited the statistical power to detect differences in some of the patients’ sociodemographic characteristics.

## Conclusion

In summary, we observed that nearly half of non-treatment seeking, non-incentivized PLWH with alcohol misuse agreed to participate in on-site CBI delivered in HIV primary care clinics. Alcohol and drug treatments have traditionally been delivered in specialized facilities, separated from care of other health conditions, making integration especially challenging. Within the context of the Affordable Care Act, there has been increasing interest in research to understand the process, cost and outcomes associated with integrating behavioural health, including alcohol treatment interventions, into general medical practice. Availability of alcohol treatment can be improved by integrating existing evidence-based approaches into clinical settings in which high-risk populations are engaged in routine care, such as HIV primary care clinics. Importantly, those patients who are most in need of care based on alcohol use and mental health severity and who have poorer HIV treatment outcomes appear to be more likely to engage in services when offered on-site in their medical homes.

## Figures and Tables

**Table 1:** Socio-demographic, clinical and behavioural characteristics and their association with CBI enrolment

	Total (n = 550)	Enrolled^a^ (n = 230)	Refused or postponed^a^ (n = 320)	p	Bivariate logistic regression OR(95%CI)	Multivariate logistic regression OR(95%CI)
Sex:						
Female	98(18%)	42(18%)	56(18%)			
Male	452(82%)	188(82%)	264(82%)	0.82		
Age: mean (SD)	43(11)	43(11)	43(11)	0.5		
Race:				0.78		
Black	259(48%)	113(49%)	146(46%)			
White		99(43%)	142(45%)			
Hispanic	24(4%)	8(4%)	16(5%)			
Others	19(4%)	8(4%)	11(3%)			
CD4 count: mean (SD)	561(306)	543(306)	573(305)	0.29		
Undetectable viral load	403(77%)	160(72%)	243(80%)	0.04	0.66(0.44,0.99)*	0.67(0.44,1.00)+
HIV med adherence:						
Very poor/poor	22(5%)	10(5%)	12(5%)			
Fair	35(7%)	12(6%)	23(8%)			
Good	53(11%)	22(11%)	31(11%)			
Very good	125(27%)	62(32%)	63(23%)			
Excellent	237(50%)	90(46%)	147(53%)	0.24		
AUDIT-C score: mean (SD)	6.32(1.98)	6.45(2.06)	6.22(1.93)	0.18		
M.I.N.I. symptom count: mean (SD)	2.51(3)	2.96(3)	2.17(3)	0.009	1.07(1.01,1.13)**	1.05(0.98,1.11)
Frequency of drinking						
Monthly or less	33(6%)	11(5%)	22(7%)			
2–4 times a month	126(23%)	53(23%)	73(23%)			
2–3 times a week	180(33%)	72(32%)	107(34%)			
4 or more time a week	208(38%)	92(40%)	116(36%)	0.66		
# of drinks on a typical drinking day						
1–4	366(67%)	150(65%)	216(68%)			
5 or 6	122(22%)	47(21%)	75(24%)			
> = 7	57(11%)	32(14%)	25(8%)	0.07		
# of drinks per week: mean (SD)	13(14)	15(15)	12(12)	0.03	1.01(1.00,1.03)*	1.10(0.99,1.02)
Frequency of binge drinking						
Daily/almost daily	44(8%)	22(10%)	22(7%)			
Weekly	137(25%)	51(22%)	86(27%)			
Monthly/Less than monthly/never	361(67%)	154(68%)	207(66%)	0.28		
Cocaine, opiates, amphetamine, or marijuana use						
Never used	422(85%)	26(12%)	48(17%)			
Past or current user	74(15%)	183(88%)	239(83%)	0.19		
Cigarette use						
Never smoked	158(29%)	66(29%)	92(29%)			
Past smoker	127(23%)	48(21%)	79(25%)			
Current smoker	257(48%)	113(50%)	144(46%)	0.51		
Mental health						
No/some panic and no depressive symptoms	247(46%)	141(62%)	162(52%)		Reference	Reference
Panic disorder or/and depressive symptoms	290(54%)	85(38%)	149(48%)	0.001	1.80(1.27,2.55)**	1.67(1.16,2.39)**

a Values and percentages may not reflect column totals for some variables because of missing data and skip patterns

+ p < 0.10

* p < 0.05

** p < 0.01.
